# Supplementary material for: Dynamic regulation of the Trypanosoma brucei transferrin receptor in response to iron starvation is mediated via the 3’UTR
Source: PLoS One. 2018 Dec 31;13(12):e0206332. doi: 10.1371/journal.pone.0206332 (PMC6312234; doi:10.1371/journal.pone.0206332)
Supplement: S1 File — Supporting information containing Figures A–C. Figure A—Sequence alignment of the BES ESAG6 3’UTRs. Figure B—Sequence alignment of the BES and putative genomic ESAG6 3’UTRs. Figure C—Extensive secondary structure is predicted in the ESAG6-3’UTR. (PDF) [file pone.0206332.s001.pdf]

Dynamic regulation of the *Trypanosoma brucei* transferrin receptor in response to iron starvation is mediated *via* the 3'UTR

Corinna Benz, Winston Lo, Nadin Fathallah, Ashley Connor-Guscott, Henry Benns, Michael D Urbaniak*

Biomedical and Life Sciences, Faculty of Health and Medicine, Lancaster University, Lancaster LA1 4YG, UK

* Corresponding author. E-mail: m.urbaniak@lancaster.ac.uk

Supplementary data

Figure A. Sequence alignment of the BES ESAG6 3'UTRs.

Figure B. Sequence alignment of the BES and putative genomic ESAG6 3'UTRs.

Figure C. Extensive secondary structure is predicted in the *ESAG6*-3'UTR.

```
BES1.ESAG6-3_UTR   GGGAAGGATGCGACCGAAACTGCGCTGCTTAGCGTGAAAGATTATGGTAATGGAGGGTTGGGAAAG-ATTGGGGAAACAA
BES2.ESAG63_UTR    AGGAAGGATGCGACAGAAGCTGCGCTGCTTAGTGTGAAAGATTATGGTAATGGAGGGTTGTGAAAG-ATTGGGGGAACAA
BES3.ESAG6-3_UTR   AGGAAGGATGCGACCGAAACTGCGCTGCTTCGCGTGAAAGATTATGGTAATGGAGGGTTGGGAAAAAATCGGTGAAACAA
BES4.ESAG6-3_UTR   AGGAAGGATGCGACAGAAGCTGCGCTGCTTAGCGTGAAAGATTATGGTAATGGAGGGTTGTGAAAG-ATTGGGGGAACAA
BES5.ESAG6-3_UTR   AGGAAGGATGCGACAGAAGCTGCGCTGCTTAGCGTGAAAGATTATGTTAATGGAGGGTTGTGAAAG-ATTGGGGGAACAA
BES7.ESAG6-3_UTR   GGGAAGGATGCGACCGAAACTGCGCTGTTTCGCGTGAATGATTATGATAATGGAGGGTTGGGAAAG-ATTGGGGGAACAA
BES10.ESAG6-3_UTR  AGGAAGGATGCGACCGAAACTGCGCTGCTTCGCGTGAAAGATTATGGTAATGGAGGGTTGGGAAAAAATCGGTGAAACAA
BES11.ESAG6-3_UTR  GGGAAGGATGCGACCGAAACTGCGCTGCTTCGCGTGAATGATTATGATAATGGAGGGTTGGGAAAAAATCGGGGAAACAA
BES12.ESAG6-3_UTR  GGGAAGGATGCGACCGAAACTGCGCTGCTTCGCGTGAAAGATTGTGGTAATGGAGGGTCGGGAAAG-ATTGGGGGAACAA
BES13.ESAG6-3_UTR  GGGAAGGATGCGACCGAAACTGCGCTGCTTAGCGTGAAAGATTATGGTAATGGAGGGTTGGGAAAG-ATTGGGGGAACAA
BES14.ESAG6-3_UTR  AGGAAGGATGCGACCGAAACTGCGCTGTTTCGCGTGAAAGATTATGGTAATGGAGGGTTGGGAAAG-ATTGGGGGAACAA
BES15.ESAG6-3_UTR  GGGAAGGATGCGACCGAAACTGCGCTGCTTAGCGTGAAAGATTATGGTAATGGAGGGTTGGGAAAG-ATTGGGGGAACAA
BES17.ESAG6-3_UTR  AGGAAGGATGCGACCGAAACTGCGCTGCTTCGCGTGAAAGATTATGGTAATGGAGGGTTGGGAAAAAATCGGTGAAACAA
                    ************* *** ******** ** * ***** **** ** *********** * ****  ** ** * *****

BES1.ESAG6-3_UTR   ATACCTATTTCTTTTATTTGGGGGAACAAATGGGCAAAAGTAACGTAAGTTTCCAGTGGGAGTGGTATGTGTGTGTGTAT
BES2.ESAG63_UTR    AAACCTATTTCTTTTATTTGGGGGAACAAATGGGCAAAAGTAACGTAAGTTTCCAGTGGGAGTGGTATGTGTGTGTGTAT
BES3.ESAG6-3_UTR   ATACCTATTTCTTTTATTTGGGGGAACAAATGGGAAATGGTAACGTAAGTTTCCAGCGGGAGTGGTATGT--GTGTGTAT
BES4.ESAG6-3_UTR   AAACCTATTTCTTTTATTTGGGGGAACAAATGGGCAAAAGTAACGTAAGTTTCCAGTGGGAGTGGTATGTGTGTGTGTAT
BES5.ESAG6-3_UTR   AAACCTATTTCTTTTATTTGGGGGAACAAATGGGCAAAAGTAACGTAAGTTTCCAGTGGGAGTGGTATGTGTGTGTGTAT
BES7.ESAG6-3_UTR   ATACCTATTTCTTTTATTTGTGGGAACAAATGGGCAGAAGTAACGTAAGTTTCTAGCGGGAGTGGTG--T--GTGTGCAT
BES10.ESAG6-3_UTR  ATACCTATTTCTTTTATTTGGGGGAACAAATGGGAAATGGTAACGTAAGTTTCCAGCGGGAGTGGTATGT--GTGTGTAT
BES11.ESAG6-3_UTR  ATACCTATTTCTTTTATTTGGGGGAACAAATGGGCAAAAGTAACGTAAGTTTCCAGCGGTAGTGGTG--T--GTGTGTAT
BES12.ESAG6-3_UTR  AAACCTATTTCTTTTATTTGTGGGAACAAATGGGAAATGGTAACGTAAGTTTCTAGCGGGAGTGGTATGT--GTGTGTAT
BES13.ESAG6-3_UTR  AAACCTATTTCTTTTATTTGGGGGAACAAATGGGCAAAAGTAACGTGAGTTTCCAGCGGGAGTGGTGTGT--GTGTGCAT
BES14.ESAG6-3_UTR  AAACCTATTTCTTTTATTTGGGGGAACAAATGGGCAAAAGTAACGTAAGTTTCCAGTGGGAGTGGTATGTGTGTGTGTAT
BES15.ESAG6-3_UTR  AAACCTATTTCTTTTATTTGGGGGAACAAATGGGCAAAAGTAACGTAAGTTTCCAGCGGGAGTGGTGTGT--GTGTGCAT
BES17.ESAG6-3_UTR  ATACCTATTTCTTTTATTTGGGGGAACAAATGGGAAATGGTAACGTAAGTTTCCAGCGGGAGTGGTATGT--GTGTGTAT
                   * ***************** ************* *    ******* ****** ** ** ******    *   ***** **

BES1.ESAG6-3_UTR   GGGGCTGGCTAAGGAAAGATGTGAGTTCGGCATGTGGTATGTACAAGCTACGAAAACGTGTGAAACAAAACGAGATGTAA
BES2.ESAG63_UTR    GGGGCTGGCTAAGGAAAGATGTGAGTTCGGCATGTGGTATGTACAAGCTACGAAAACGTGTGAAACAAAACGAGATGTAA
BES3.ESAG6-3_UTR   GGGGCTGGCTAAGGAAAGATGTAAGTTCGGCATGTGGTATGTACAAGCTATGAAAACGAGTGAAACAAAACGAGTTATAA
BES4.ESAG6-3_UTR   GGGGCTGGCTAAGGAAAGATGTGAGTTCGGCATGTGGTATGTACAAGCTACGAAAACGTGTGAAACAAAACGAGATGTAA
BES5.ESAG6-3_UTR   GGGGCTGGCTAAGGAAAGATGTGAGTTCGGCATGTGGTATGTACAAGCTACGAAAACGTGTGAAATAAAACGAGATGTAA
BES7.ESAG6-3_UTR   GGGACGGGTTAAGGAAAGATGTATGTTCGGCATGTGGTATGTACAAGCTACGAAAACGTTTGAAACAAAACGAGATGTAA
BES10.ESAG6-3_UTR  GGGGCTGGCTAAGGAAAGATGTAAGTTCGGCATGTGGTATGTACAAGCTATGAAAACGAGTGAAACAAAACGAGTTATAA
BES11.ESAG6-3_UTR  GGGGCTGGCTAAGGAAAGATGTAAGTTCGGCATGTGGTATATACAAGCTACGAAAACGTGTGAAACAAAACGAGATGTAA
BES12.ESAG6-3_UTR  GGGGCTGGCTAAGGAAAGATGTAAGTTCGGCATGTGGTATGTACAAGCTACGAAAACGTGTGAAACAAAACGATATGTAA
BES13.ESAG6-3_UTR  GGGGCTGACTAAGGAAAGATGTAAGTTCGGAATGTGGTATGTACAAGCTACGAAAACGTGTGAAACAAAACGATATGTAA
BES14.ESAG6-3_UTR  GGGGCTGGCTAAGGAAAGATGTAAGTTCGGAATGTGGTATGTACAAGCTACGAAAACGTTTGAAACAAAACGAGATGTAA
BES15.ESAG6-3_UTR  GGGACGGGTTAAGGAAAGATGTAAGTTCGGCATGTGGTATGTACAAGCTACGAAAACGTTTGAAACAAAACGAGATGTAA
BES17.ESAG6-3_UTR  GGGGCTGGCTAAGGAAAGATGTAAGTTCGGCATGTGGTATGTACAAGCTATGAAAACGAGTGAAACAAAACGAGTTATAA
                   *** * *  *************  ****** ********* ********* *******  ***** *******  * ***

BES1.ESAG6-3_UTR   GGGGAAAATGTAACAACCAACTATGTTAAATT-TCAG--GAGACTATTTTTCAAATTTAGTTACAACAAAGTAAATGTCA
BES2.ESAG63_UTR    GGGGAAAATGTAACAACCAACTATGTTAAATT-TCAG--GAGACTATTTTTCAAATTTAGTTACAACAAAGTAAATGTCA
BES3.ESAG6-3_UTR   GGGGGAAATGTAACAACCAACTTTGCTAAATTTTCAG--GATACTATTTTTCAAATTTAGTTACAACAAAGTAAATGTCA
BES4.ESAG6-3_UTR   GGGGAAAATGTAACAACCAACTATGTTAAATT-TCAG--GAGACTATTTTTCAAATTTAGTTACAACAAAGTAAATGTCA
BES5.ESAG6-3_UTR   GGGGAAAATGTAACAACCAACTATGTTAAATT-TCAG--GAGACTATTTTTCAAATTTAGTTACAACAAAGTAAATGTCA
BES7.ESAG6-3_UTR   GGGGAAAATGTAACAACTAACTATGTTAAATT-TCAG--GAGACTATTTTTCAAATTTAGTTACAACAAAGTAAATGTCA
BES10.ESAG6-3_UTR  GGGGGAAATGTAACAACCAACTATGTTAAATT-TTAG--GATACTATTTTTCAAATTTAGTTACAACAAAGTAAATGTCA
BES11.ESAG6-3_UTR  GGGGAAAATGTAACAACCAACTATGTTAAATT-TCAG--GAGACTATTTTTCAAATTTAGTTACAACACAGTAAATGTTA
BES12.ESAG6-3_UTR  GGGGAAAATGTAACAACCAACTATGTTAAATT-TCAG--GAGACTATTTTTCAAATTTAGTTACAACAAAGTAAATGTCA
BES13.ESAG6-3_UTR  GGGGAAAATGTAACAACCAACTATGTTAAATT-TCAA--GAGACTATTTTTCAAATTTAGTTACAACAAAGTAAATGTCA
BES14.ESAG6-3_UTR  GGGGAAAATGTAACAACCAACTATGTTAAATT-TTAG--GAGACTATTTTTCAAATTTAGTTACAACAAAGTAAATGTCA
BES15.ESAG6-3_UTR  GGGGAAAATGTAACAACTATCTATGTTAAATT-TCAGAAGAGACTATTTTTCAAATTTAGTTACAACAAAGTAAATGTCA
BES17.ESAG6-3_UTR  GGGGGAAATGTAACAACCAACTTTGCTAAATTTTCAG--GATACTATTTTTCAAATTTAGTTACAACAAAGTAAATGTCA
                   **** ************ * ** ** ****** * *   ** ************************** ********* *

                       ▼            ▼     ▼
BES1.ESAG6-3_UTR   AATAATGCCAACTATAAAGGAAAA---------
BES2.ESAG63_UTR    AATAATGCCAACTATAAAGGAAAACTAG---AC
BES3.ESAG6-3_UTR   AATAAAGCCAACTATAAAAGAAAACTAG----A
BES4.ESAG6-3_UTR   AATAATGCCAACTATAAAGGAAAACTAG----A
BES5.ESAG6-3_UTR   AATAATGCCAACTATAAAGGAAAACTAG----A
BES7.ESAG6-3_UTR   AATAATGCCAACTATAAAGGAAAACTAGACTAA
BES10.ESAG6-3_UTR  AATAATGCCAACTATAAAAGAAAACTAGA---C
BES11.ESAG6-3_UTR  AATAATGCCAACTATAAAGGAAAACCAGAC-TA
BES12.ESAG6-3_UTR  AATAATGCCAACTATAAAGGAAAACTAGA--CT
BES13.ESAG6-3_UTR  AATAATGCCAACTATAAAGGAAAACTAGA--CT
BES14.ESAG6-3_UTR  AATAATGCCAACTATAAAGGAAAACTAG----A
BES15.ESAG6-3_UTR  AATAATGCCAACTATAAAGGAAAACTAG----A
BES17.ESAG6-3_UTR  AATAATGCCAACTATAAAAGAAAACTAG----A
                   ***** ************ *****
```

Figure A. Sequence alignment of the telomeric BES ESAG6 3'UTRs. The observed polyadenylation sites are indicated with triangles above the sequence, and sequence identity indicated by stars below the sequence. Sequences immediately downstream of the stop codon were aligned with T-Coffee [1].

```
BES1.ESAG6-3_UTR     GGGAAGGATGCGACCGAAACTGCGCTGCTTAGCGTGAAAGATTATGGTAATGGAGGGTTGGGAAA-GATTGGGGAAACAA
BES2.ESAG63_UTR      AGGAAGGATGCGACAGAAGCTGCGCTGCTTAGTGTGAAAGATTATGGTAATGGAGGGTTGTGAAA-GATTGGGGGAACAA
BES3.ESAG6-3_UTR     AGGAAGGATGCGACCGAAACTGCGCTGCTTCGCGTGAAAGATTATGGTAATGGAGGGTTGGGAAAAAATCGGTGAAACAA
BES4.ESAG6-3_UTR     AGGAAGGATGCGACAGAAGCTGCGCTGCTTAGCGTGAAAGATTATGGTAATGGAGGGTTGTGAAA-GATTGGGGGAACAA
BES5.ESAG6-3_UTR     AGGAAGGATGCGACAGAAGCTGCGCTGCTTAGCGTGAAAGATTATGTTAATGGAGGGTTGTGAAA-GATTGGGGGAACAA
BES7.ESAG6-3_UTR     GGGAAGGATGCGACCGAAACTGCGCTGTTTCGCGTGAATGATTATGATAATGGAGGGTTGGGAAA-GATTGGGGGAACAA
BES10.ESAG6-3_UTR    AGGAAGGATGCGACCGAAACTGCGCTGCTTCGCGTGAAAGATTATGGTAATGGAGGGTTGGGAAAAAATCGGTGAAACAA
BES11.ESAG6-3_UTR    GGGAAGGATGCGACCGAAACTGCGCTGCTTCGCGTGAATGATTATGATAATGGAGGGTTGGGAAAAAATCGGGGAAACAA
BES12.ESAG6-3_UTR    GGGAAGGATGCGACCGAAACTGCGCTGCTTCGCGTGAAAGATTGTGGTAATGGAGGGTCGGGAAA-GATTGGGGGAACAA
BES13.ESAG6-3_UTR    GGGAAGGATGCGACCGAAACTGCGCTGCTTAGCGTGAAAGATTATGGTAATGGAGGGTTGGGAAA-GATTGGGGGAACAA
BES14.ESAG6-3_UTR    AGGAAGGATGCGACCGAAACTGCGCTGTTTCGCGTGAAAGATTATGGTAATGGAGGGTTGGGAAA-GATTGGGGGAACAA
BES15.ESAG6-3_UTR    GGGAAGGATGCGACCGAAACTGCGCTGCTTAGCGTGAAAGATTATGGTAATGGAGGGTTGGGAAA-GATTGGGGGAACAA
BES17.ESAG6-3_UTR    AGGAAGGATGCGACCGAAACTGCGCTGCTTCGCGTGAAAGATTATGGTAATGGAGGGTTGGGAAAAAATCGGTGAAACAA
Tb927.9.15680-3_UTR  TGT--GAATGTAAAAGAAGTA--ACTGGCTCGTATAT-ACATTGATCTCTTG---TATTGGGTAGAGCTCG-CCATTTTG
                      *   * ***  *  ***      ***  * *  *     ***    *  **     * * * *    * *

BES1.ESAG6-3_UTR     ATACCTATTTCTTT---TATTTGGGGGAACAAATGGGCAAAAGTAACG--TAAGTTTCCAGTGGGAGTGGTATGTGTGTG
BES2.ESAG63_UTR      AAACCTATTTCTTT---TATTTGGGGGAACAAATGGGCAAAAGTAACG--TAAGTTTCCAGTGGGAGTGGTATGTGTGTG
BES3.ESAG6-3_UTR     ATACCTATTTCTTT---TATTTGGGGGAACAAATGGGAAATGGTAACG--TAAGTTTCCAGCGGGAGTGGTATGT--GTG
BES4.ESAG6-3_UTR     AAACCTATTTCTTT---TATTTGGGGGAACAAATGGGCAAAAGTAACG--TAAGTTTCCAGTGGGAGTGGTATGTGTGTG
BES5.ESAG6-3_UTR     AAACCTATTTCTTT---TATTTGGGGGAACAAATGGGCAAAAGTAACG--TAAGTTTCCAGTGGGAGTGGTATGTGTGTG
BES7.ESAG6-3_UTR     ATACCTATTTCTTT---TATTTGTGGGAACAAATGGGCAGAAGTAACG--TAAGTTTCTAGCGGGAGTGGTG--T--GTG
BES10.ESAG6-3_UTR    ATACCTATTTCTTT---TATTTGGGGGAACAAATGGGAAATGGTAACG--TAAGTTTCCAGCGGGAGTGGTATGT--GTG
BES11.ESAG6-3_UTR    ATACCTATTTCTTT---TATTTGGGGGAACAAATGGGCAAAAGTAACG--TAAGTTTCCAGCGGTAGTGGTG--T--GTG
BES12.ESAG6-3_UTR    AAACCTATTTCTTT---TATTTGTGGGAACAAATGGGAAATGGTAACG--TAAGTTTCTAGCGGGAGTGGTATGT--GTG
BES13.ESAG6-3_UTR    AAACCTATTTCTTT---TATTTGGGGGAACAAATGGGCAAAAGTAACG--TGAGTTTCCAGCGGGAGTGGTGTGT--GTG
BES14.ESAG6-3_UTR    AAACCTATTTCTTT---TATTTGGGGGAACAAATGGGCAAAAGTAACG--TAAGTTTCCAGTGGGAGTGGTATGTGTGTG
BES15.ESAG6-3_UTR    AAACCTATTTCTTT---TATTTGGGGGAACAAATGGGCAAAAGTAACG--TAAGTTTCCAGCGGGAGTGGTGTGT--GTG
BES17.ESAG6-3_UTR    ATACCTATTTCTTT---TATTTGGGGGAACAAATGGGAAATGGTAACG--TAAGTTTCCAGCGGGAGTGGTATGT--GTG
Tb927.9.15680-3_UTR  CAGCCTATGCGTGCCACAACATCTGTCACCGTGTGTA-ACAAGTGAAGTTCCAGTTTACCTTGGTCATTGCATTT--AAC
                        *****   *      *  *  *  * *   **   *   ** * *    *****     **   * *    *

BES1.ESAG6-3_UTR     TGT-ATGGGGCTGGCTAAGGAA-AGATGTGAGTTCGGCATGTGGTA-TGT-ACAAGCTACGAAAACGTGTGAAACAAAAC
BES2.ESAG63_UTR      TGT-ATGGGGCTGGCTAAGGAA-AGATGTGAGTTCGGCATGTGGTA-TGT-ACAAGCTACGAAAACGTGTGAAACAAAAC
BES3.ESAG6-3_UTR     TGT-ATGGGGCTGGCTAAGGAA-AGATGTAAGTTCGGCATGTGGTA-TGT-ACAAGCTATGAAAACGAGTGAAACAAAAC
BES4.ESAG6-3_UTR     TGT-ATGGGGCTGGCTAAGGAA-AGATGTGAGTTCGGCATGTGGTA-TGT-ACAAGCTACGAAAACGTGTGAAACAAAAC
BES5.ESAG6-3_UTR     TGT-ATGGGGCTGGCTAAGGAA-AGATGTGAGTTCGGCATGTGGTA-TGT-ACAAGCTACGAAAACGTGTGAAATAAAAC
BES7.ESAG6-3_UTR     TGC-ATGGGACGGGTTAAGGAA-AGATGTATGTTCGGCATGTGGTA-TGT-ACAAGCTACGAAAACGTTTGAAACAAAAC
BES10.ESAG6-3_UTR    TGT-ATGGGGCTGGCTAAGGAA-AGATGTAAGTTCGGCATGTGGTA-TGT-ACAAGCTATGAAAACGAGTGAAACAAAAC
BES11.ESAG6-3_UTR    TGT-ATGGGGCTGGCTAAGGAA-AGATGTAAGTTCGGCATGTGGTA-TAT-ACAAGCTACGAAAACGTGTGAAACAAAAC
BES12.ESAG6-3_UTR    TGT-ATGGGGCTGGCTAAGGAA-AGATGTAAGTTCGGCATGTGGTA-TGT-ACAAGCTACGAAAACGTGTGAAACAAAAC
BES13.ESAG6-3_UTR    TGC-ATGGGGCTGACTAAGGAA-AGATGTAAGTTCGGAATGTGGTA-TGT-ACAAGCTACGAAAACGTGTGAAACAAAAC
BES14.ESAG6-3_UTR    TGT-ATGGGGCTGGCTAAGGAA-AGATGTAAGTTCGGAATGTGGTA-TGT-ACAAGCTACGAAAACGTTTGAAACAAAAC
BES15.ESAG6-3_UTR    TGC-ATGGGACGGGTTAAGGAA-AGATGTAAGTTCGGCATGTGGTA-TGT-ACAAGCTACGAAAACGTTTGAAACAAAAC
BES17.ESAG6-3_UTR    TGT-ATGGGGCTGGCTAAGGAA-AGATGTAAGTTCGGCATGTGGTA-TGT-ACAAGCTATGAAAACGAGTGAAACAAAAC
Tb927.9.15680-3_UTR  TTTCCTAAACCT--CCATCCACATCGTGC-ACTCCGCCAAACTGTAGCGCGGGAAGTTGTTGAAAAATGTGAATCTCATT
                     *    *    *     *   *     **    * **  *    ***       *** *    ***    ****    *

BES1.ESAG6-3_UTR     GA-----GATGTAAGGGGAAAATGTAACAACCAACTATGTTAAATT-TCAG--GAGACTATTTTTCAAATT----TAGTT
BES2.ESAG63_UTR      GA-----GATGTAAGGGGAAAATGTAACAACCAACTATGTTAAATT-TCAG--GAGACTATTTTTCAAATT----TAGTT
BES3.ESAG6-3_UTR     GA-----GTTATAAGGGGGAAATGTAACAACCAACTTTGCTAAATTTTCAG--GATACTATTTTTCAAATT----TAGTT
BES4.ESAG6-3_UTR     GA-----GATGTAAGGGGAAAATGTAACAACCAACTATGTTAAATT-TCAG--GAGACTATTTTTCAAATT----TAGTT
BES5.ESAG6-3_UTR     GA-----GATGTAAGGGGAAAATGTAACAACCAACTATGTTAAATT-TCAG--GAGACTATTTTTCAAATT----TAGTT
BES7.ESAG6-3_UTR     GA-----GATGTAAGGGGAAAATGTAACAACTAACTATGTTAAATT-TCAG--GAGACTATTTTTCAAATT----TAGTT
BES10.ESAG6-3_UTR    GA-----GTTATAAGGGGGAAATGTAACAACCAACTATGTTAAATT-TTAG--GATACTATTTTTCAAATT----TAGTT
BES11.ESAG6-3_UTR    GA-----GATGTAAGGGGAAAATGTAACAACCAACTATGTTAAATT-TCAG--GAGACTATTTTTCAAATT----TAGTT
BES12.ESAG6-3_UTR    GA-----TATGTAAGGGGAAAATGTAACAACCAACTATGTTAAATT-TCAG--GAGACTATTTTTCAAATT----TAGTT
BES13.ESAG6-3_UTR    GA-----TATGTAAGGGGAAAATGTAACAACCAACTATGTTAAATT-TCAA--GAGACTATTTTTCAAATT----TAGTT
BES14.ESAG6-3_UTR    GA-----GATGTAAGGGGAAAATGTAACAACCAACTATGTTAAATT-TTAG--GAGACTATTTTTCAAATT----TAGTT
BES15.ESAG6-3_UTR    GA-----GATGTAAGGGGAAAATGTAACAACTATCTATGTTAAATT-TCAGAAGAGACTATTTTTCAAATT----TAGTT
BES17.ESAG6-3_UTR    GA-----GTTATAAGGGGGAAATGTAACAACCAACTTTGCTAAATTTTCAG--GATACTATTTTTCAAATT----TAGTT
Tb927.9.15680-3_UTR  GCATCCTTTTGCAATCCGACTTTCTT-GGTCCACGCAAG-CATGGG-TCAG--AAAGCATTTTAGCCAACCTCGGAAGTC
                     *        *  **   *    * *     * *     *  *     * *    *  *  ***  * **       ***

BES1.ESAG6-3_UTR     ACAACAAAGTAAATGTCAAATAATGCCAACTAT--AAAGGAAA---------A
BES2.ESAG63_UTR      ACAACAAAGTAAATGTCAAATAATGCCAACTAT--AAAGGAAAACTAG---AC
BES3.ESAG6-3_UTR     ACAACAAAGTAAATGTCAAATAAAGCCAACTAT--AAAAGAAAACTAG----A
BES4.ESAG6-3_UTR     ACAACAAAGTAAATGTCAAATAATGCCAACTAT--AAAGGAAAACTAG----A
BES5.ESAG6-3_UTR     ACAACAAAGTAAATGTCAAATAATGCCAACTAT--AAAGGAAAACTAG----A
BES7.ESAG6-3_UTR     ACAACAAAGTAAATGTCAAATAATGCCAACTAT--AAAGGAAAACTAGACTAA
BES10.ESAG6-3_UTR    ACAACAAAGTAAATGTCAAATAATGCCAACTAT--AAAAGAAAACTAGA---C
BES11.ESAG6-3_UTR    ACAACACAGTAAATGTTAAATAATGCCAACTAT--AAAGGAAAACCAGAC-TA
BES12.ESAG6-3_UTR    ACAACAAAGTAAATGTCAAATAATGCCAACTAT--AAAGGAAAACTAGA--CT
BES13.ESAG6-3_UTR    ACAACAAAGTAAATGTCAAATAATGCCAACTAT--AAAGGAAAACTAGA--CT
BES14.ESAG6-3_UTR    ACAACAAAGTAAATGTCAAATAATGCCAACTAT--AAAGGAAAACTAG----A
BES15.ESAG6-3_UTR    ACAACAAAGTAAATGTCAAATAATGCCAACTAT--AAAGGAAAACTAG----A
BES17.ESAG6-3_UTR    ACAACAAAGTAAATGTCAAATAATGCCAACTAT--AAAAGAAAACTAG----A
Tb927.9.15680-3_UTR  ACAACCAAGGAACTCATAAAAAGAGCTCCTTCCTCCCTGGTA--C--A----G
                     *****  ** ** *   *** *  **    *        * *
```

Figure B. Sequence alignment of the BES and putative non-telomeric ESAG6 3'UTRs. The sequence identity is indicated by stars below the sequence. Sequences immediately downstream of the stop codon were aligned with T-Coffee [1]

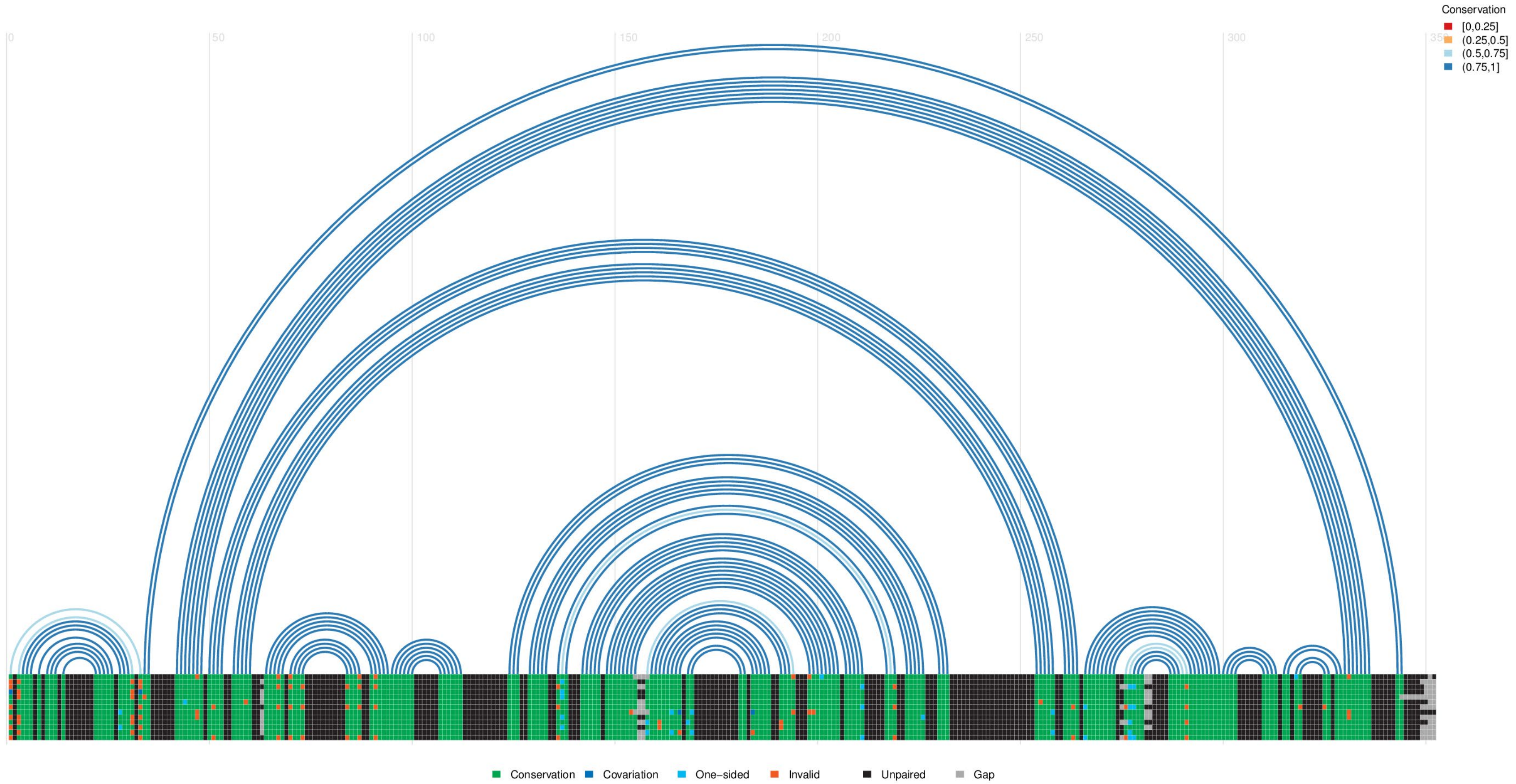

Figure C. Extensive secondary structure is predicted in the *ESAG6*-3'UTR. Simultaneous folding and alignment of the 13 *ESAG6* 3'UTRs was performed using LocARNA [2], with the predicted minimum free energy structure of the consensus sequence visualised using R-chie [3]. Arcs indicate the predicted base-pairing, coloured by the degree of sequence conservation. The coloured blocks at the base represent the alignment of the 13 *ESAG6* 3'UTRs coloured by the structural covariance.

References:

1. Notredame C, Higgins D, Heringa J. T-Coffee: A novel method for multiple sequence alignment. J Mol Biol. 2000;302:205-17.
2. Will S, Joshi T, Hofacker IL, Stadler PF, Backofen R. LocARNA-P: accurate boundary prediction and improved detection of structural RNAs. RNA. 2012;18(5):900-14. Epub 2012/03/28. doi: 10.1261/rna.029041.111. PubMed PMID: 22450757; PubMed Central PMCID: PMCPMC3334699.
3. Lai D, Proctor JR, Zhu JY, Meyer IM. R-CHIE: a web server and R package for visualizing RNA secondary structures. Nucleic Acids Res. 2012;40(12):e95. Epub 2012/03/22. doi: 10.1093/nar/gks241. PubMed PMID: 22434875; PubMed Central PMCID: PMCPMC3384350.
